# Supplementary material for: Identification and Characterization of Two Novel Noda-like Viruses from Rice Plants Showing the Dwarfing Symptom
Source: Viruses. 2022 May 27;14(6):1159. doi: 10.3390/v14061159 (PMC9231309; doi:10.3390/v14061159)
Supplement: Supplementary file 1 [file viruses-14-01159-s001.zip › viruses-1729154-supplementary.pdf]

viruses

MDPI

Supplementary Materials

Identification and Characterization of Two Novel Noda-Like Viruses from Rice Plants Showing Dwarf Symptoms

Table S1. Primers used in this study.

Primer	Sequence 5′ > 3′
Contig3-detection-F	ATGGAGACGCCCAACTGATG
Contig3-detection-R	TCTCCAACGCGATAGACAGC
Contig15-detection-F	GCCCTAGCATATCAAGTCTC
Contig15-detection-R	AATTATTTGTGTGCTGTATTTGATC
Contig3206-detection-F	CCGTCGTGGACGTACCATAG
Contig3206-detection-R	ACGAACTGCTTGCGGTTTTC
Contig15-5RACEGSP1-R	GCATTGTGTGGTAATTCAGGG
Contig15-5RACEGSP2-R	AGCCTGCTTCTTCGGCTATC
Contig15-5RACEGSP3-R	GCTATCAACCCAGCGTAGCG
Contig3-5RACEGSP1-R	GTGTAACTTAGGACGCGTTCG
Contig3-5RACEGSP2-R	GTTCCAAGTTGTTGGGGTCG
Contig3-5RACEGSP3-R	CGGATGAAGTATGCGAAGAG
Contig15-3RACEGSP1-F	GGGCACGTAAGGTGCTTGCG
Contig15-3RACEGSP2-F	CAGCCGTCAGAAGTGGGGTA
Contig15-3RACEGSP3-F	GCCCTAGCATATCAAGTCTC
Contig3-3RACEGSP1-F	GAACGCCGAGCTGTGCAAG
Contig3-3RACEGSP2-F	GTTTGGCTACGTGGTTGGTCC
Contig3-3RACEGSP3-F	GCATAGAACCACATGCATGG
RNLV1 RNA1-Junction-F	GGAAGCTACCGCACGAGAATG
RNLV1 RNA1-Junction-R	GCGCAACACGACATCTAGGG
RNLV1 RNA2-Junction-F	CGCAAGAAGGATAGAGCCGC
RNLV1 RNA2-Junction-R	CCGAGCTCCATAGAGCTGTAC
RNLV1 RNA3-Junction-F	CACTAACTAGTGAACGCTAGTGATAG
RNLV1 RNA3-Junction-R	GGCTTGATTGTTGGTGAGCTG
RNLV1 RNA1-Full-F	GATATATTATCTTTATTTTAAACAAATAATC
RNLV1 RNA1-Full-R	GCCGGTGTTAAAGTGAT
RNLV1 RNA2-Full-F	GATATATTATCCTATTAACATGCCTAGACGC
RNLV1 RNA2-Full-R	GCCAGTACATGGGGGTTTA
Contig3545-5RACEGSP1-R	GACGAGCGCACGGTTCTCCATATTCGTC
Contig3545-5RACEGSP2-R	GGGCGAGTCATCATCGATGGAACGATGG
Contig3206-5RACEGSP1-R	AGAAGTGCCCAGGGGAGTACTCCC
Contig3206-5RACEGSP2-R	TGATCCGCTGGTATGACGAGGCGAATCG
Contig3206-5RACEGSP3-R	ACGACCCATGGCAACGGGCGCTGACGTC
Contig3545-3RACEGSP1-F	CGGACCACCGCGACGTCCTTCCAGGA
Contig3545-3RACEGSP2-F	AAGTAGCGCAGAAGGTGGAGGAGCTGCT
Contig3545-3RACEGSP3-F	AGGTGGTGGCGTTCCTCAAGGGGGAGGA
Contig3206-3RACEGSP1-F	ATATTAGTAAGTACCCCCGACCCCGCGG
Contig3206-3RACEGSP2-F	CTTCCCTAACGACCCCGAGGCGTTAAAC
RNLV2 RNA1-Full-F	GGTTTTGTTACTTTCCTGTTGTTG
RNLV2 RNA1-Full-R	ACCGTTCTAAATAGAAAATAATAATTC
RNLV2 RNA2-Full-F	GGTATTGTTTTCCCCATAACATCGTG
RNLV2 RNA2-Full-R	AGAAATTTCTAATGTCCGGTCCAG
pGD-RNLV1 B2-F	tacaaatctatctctggatccATGAACAAAACAATCAACATGGCG
pGD-RNLV1 B2-R	gtttgaacgagctctgtcgacCTATTCCGAATCATCATTCTCGTG
pGD-RNLV2 B2-F	tacaaatctatctctggatccATGAACAAAACAATCAACATGGCG
pGD-RNLV2 B2-R	gtttgaacgagctctgtcgacCTATTCCGAATCATCATTCTCGTG
pGR106-RNLV1 B2-F	tcagcaccagctagcatcgatATGAACAAAACAATCAACATGGCG
pGR106-RNLV1 B2-R	aaccgttcatcggcggtcgacCTATTCCGAATCATCATTCTCGTG
pGR106-RNLV2 B2-F	tcagcaccagctagcatcgatATGCCGTCGACATACGAGCA
pGR106-RNLV2 B2-R	aaccgttcatcggcggtcgacCTACAACGAAAAGCAATAAAGCGC
pCB301-RNLV1 RNA1-F	tttcatttggagaggATATATTATCTTTATTTTAAACAAATAATC
pCB301-RNLV1 RNA1-R	atgccatgccgacccGCCGGTGTTAAAGTGAT
pCB301-RNLV1 RNA1-B2 backbone-F	TAGATCAAATACAGCACACAAATAATTTC

pCB301-RNLV1 RNA1-B2 backbone-R	TTCCGAATCATCATTCTCGTGC
pCB301-RNLV1 RNA1-B2:GFP-F	ACGAGAATGATGATTCGGAAATGGTGAGCAAGGGCGACG
pCB301-RNLV1 RNA1-B2:GFP-R	TGTGTGCTGTATTTGATCTACTTGTACAGCTCGTCCATGCC
pCB301-RNLV1 RNA1-ORF1a backbone-F	ATGAACAAAACAATCAACATGGCG
pCB301-RNLV1 RNA1-ORF1a backbone-R	TGGTTATAAGTTGAAAAGGTTAATAGTGAA
pCB301-RNLV1 ORF1a opt-F	ACCTTTTCAACTTATAACCAATGAAATCTAACATGAAGATCATGTGG
pCB301-RNLV1 ORF1a opt-R	ATGTTGATTGTTTTGTTCATTGATATGAACGGCCTCGATTACC
pCB301-RNLV1 RNA2-F	tttcatttggagaggATATATTATCCTATTAACATGCCTAGACGC
pCB301-RNLV1 RNA2-R	atgccatgccgacccGCCAGTACATGGGGGTTTA
RNLV1 RNA1 592-F	GAAGAACACGTAGCAGCTGA
RNLV1 RNA1 592-R	CTCGTGCGGTAGCTTCCCTT
T7-RNLV1 RNA1-F	GATCACTAATACGACTCACTATAGATATATTATCTTTATTTTAAACAAATAATC
T7-RNLV1 RNA1-R	GCCGGTGTTAGAGTGATATATTA
T7-RNLV1 RNA2-F	GATCACTAATACGACTCACTATAGATATATTATCCTATTAACATGCCTAG
T7-RNLV1 RNA2-R	GCCAGTACATGGGGGTTTAAATA
RNLV1 RNA1 422-F	ACCCTAGATGTCGTGTTGCG
RNLV1 RNA1 422-R	GCAGAATTTGGGTGGCTGTG
RNLV1 RNA2 509-F	TTGCAGATCAGGGACGAGTG
RNLV1 RNA2 509-R	ACTTTGGTCAGAGCCTGGTG
pET-28a-RNLV1 CP-F	cagcaaatgggtcgcggatccATGCCTAGACGCAACAAGTCAA
pET-28a-RNLV1 CP-R	tgcggccgcaagcttgtcgacTTAGCGATATATTATCCGGTTTAGTCC
pFASTBAC-RNLV1 CP-F	cccaccatcgggcgcggatccATGCCTAGACGCAACAAGTCAA
pFASTBAC-RNLV1 CP-F	gcgactagtgagctcgtcgacTTAGCGATATATTATCCGGTTTAGTCC
RNLV1 CP N480A-F	CTGATTACgcTGAGCGTAACAAGCTCTGGGGGA
RNLV1 CP N480A-R	TACGCTCAgcGTAATCAGCAGGCCATGCATGTG
pGD-RNLV1 CP-F	tacaaatctatctctggatccATGCCTAGACGCAACAAGTCAA
pGD-RNLV1 CP-R	gtttgaacgagctctgtcgacTTAGCGATATATTATCCGGTTTAGTCC

Table S2. Summary of conserved domain prediction of capsid proteins from RNLV1, RNLV2, and other related viruses.

Protein Accession No.	Virus Species	Host	Family	Genus	Domain Accession	Domain	Region
NC_004144	Flock House virus	Insect	*Nodaviridae*	*Alphanodavirus*	cl03372	Peptidase_A6	53-363
NC_002691	Nodamura virus	Insect	*Nodaviridae*	*Alphanodavirus*	cl03372 (pfam01829)	Peptidase_A6	42-355
NC_008041	Redspotted grouper nervous necrosis virus	Fish	*Nodaviridae*	*Betanodavirus*	cl29941	Viral_coat	1-338
NC_003449	Striped jack nervous necrosis virus	Fish	*Nodaviridae*	*Betanodavirus*	cl29941 (pfam11729)	Viral_coat (Capsid-VNN)	1-340
EF137180	Penaeus vannamei nodavirus	Shrimp	*Nodaviridae*	Unclassified	cl29941	Viral_coat	99-197
MT270123	Covert mortality nodavirus	Shrimp	*Nodaviridae*	Unclassified	cl29941	Viral_coat	19-228
HM030973	Santeuil virus	Nematode	*Nodaviridae*	Unclassified	cl29941	Viral_coat	2-178
HM030971	Orsay virus	Nematode	*Nodaviridae*	Unclassified	cl29941	Viral_coat	10-220
KJ632943	Mosinovirus	Insect	Nodaviridae	Unclassified	cl04161	Peptidase_A21	190-442
KR003800	Lutzomyia nodavirus	Insect	*Nodaviridae*	Unclassified	cl04161	Peptidase_A21	66-558
NC_035466	Lake Sinai virus 1	Insect	*Sinhaliviridae*	*Sinaivirus*	cl04161	Peptidase_A21	87-318; 437-505
NC_035467	Lake Sinai virus 2	Insect	*Sinhaliviridae*	*Sinaivirus*	cl04161	Peptidase_A21	392-460
NC_010712	Chronic bee paralysis virus	Insect	Unclassified	Unclassified	cl24918 (pfam16506)	virion glycoprotein	273-348
NC_001990	Nudaurelia capensis beta virus	Insect	*Alphatetraviridae*	*Betatetraviridae*	cl04161	Peptidase_A21	1-536
NC_038335	Nudaurelia capensis omega virus	Insect	*Alphatetraviridae*	*Omegatetravirus*	cl04161	Peptidase_A21	1-570
NC_014126	Providence virus	Insect	*Carmotetravirus*	*Alphacarmotetravirus*	cl04161	Peptidase_A21	54-679
NC_003412	Euprosterna elaeasa virus	Insect	*Permutotetraviridae*	*Alphapermutotetravirus*	cl04161	Peptidase_A21	201-694
NC_043231	Thosea asigna virus	Insect	*Permutotetraviridae*	*Alphapermutotetravirus*	cl04161	Peptidase_A21	201-695
ON260789	Rrice-associated noda-like virus 1	Plant	Unclassified	Unclassified	cl04161	Peptidase_A21	113-289; 329-480
ON260787	Rice-associated noda-like virus 2	Plant	Unclassified	Unclassified	cl29941	Viral_coat	91-260

MK533153	Tetranychus urticae-associated nodavirus 2	Insect	Unclassified	Unclassified	cl04161	Peptidase_A21	8-257
NC_033308	Hubei noda-like virus 9	Insect	Unclassified	Unclassified	cl04161	Peptidase_A21	16-280

Table S3. Viruses used for protein homology and phylogenetic analyses.

Virus Species	RdRp Accession No.	CP Accession No.
Black beetle virus	NC_001411	NC_002037
Boolarra virus	NC_004142	NC_004145
Flock House virus	NC_004146	NC_004144
Nodamura virus	NC_002690	NC_002691
Pariacoto virus	NC_003691	NC_003692
Barfin flounder nervous necrosis virus	NC_013458	NC_013459
Redspotted grouper nervous necrosis virus	NC_008040	NC_008041
Striped jack nervous necrosis virus	NC_003448	NC_003449
Tiger puffer nervous necrosis virus	NC_013460	NC_013461
Macrobrachium rosenbergii nodavirus	AY231436	AY222840
Penaeus vannamei nodavirus	HQ259079	EF137180
Covert mortality nodavirus	MT270124	MT270123
Wuhan nodavirus	AY962576	DQ233638
Le Blanc virus	JQ943578	JQ943579
Santeuil virus	HM030972	HM030973
Orsay virus	HM030970	HM030971
Mosinovirus	KJ632942	KJ632943
Lutzomyia nodavirus	KR003799	KR003800
Bat guano associated nodavirus	HM228873	-
Lake Sinai virus 1	NC_035466	NC_035466
Lake Sinai virus 2	NC_035467	NC_035467
Chronic bee paralysis virus	NC_010711	NC_010712
Nudaurelia capensis beta virus	NC_001990	NC_001990
Helicoverpa armigera stunt virus	NC_001981	NC_001982
Dendrolimus punctatus virus	NC_005898	NC_005899
Nudaurelia capensis omega virus	-	NC_038335
Providence virus	NC_014126	NC_014126
Euprosterna elaeasa virus	NC_003412	NC_003412
Thosea asigna virus	NC_043232	NC_043231

-, sequences not available.

Table S4. Summary of RNA-seq.

Project	Total Reads (150 nt)	Mapping Genome	Non-host Reads	Assembled Contigs (> 200 nt)
2016-ZJ	62,652,118	Oryza sativa	4,587,386	49,035
2019-SH	78,856,691	Oryza sativa	5,898,480	52,647

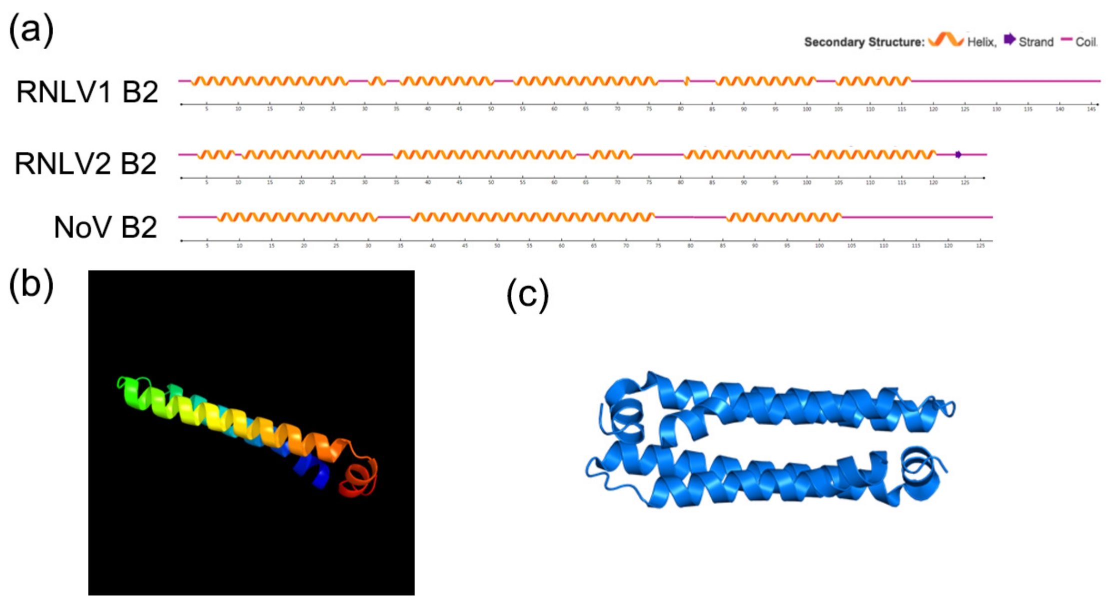

Figure S1. Protein Structure of RNLV1 and RNLV2 B2 proteins (**a**) Predicted secondary structure of B2 proteins of RNLV1, RNLV2, and NoV using SurfP2.0. (**b**) Predicted protein model of RNLV2 B2 generated by Phery2 based on the NoV B2 model by Phyre2.0. The model of NLRV2 B2 was coloured by rainbow N - C terminus (purple-red). (**c**) Model of Nov B2 dimer (3g80) from the Protein Data Bank (PDB).

ATGAAATCTAACATGAAGATCATGTGGTCCAAAAGGCCGAAGCTAGACAAACTGGAGTGCTCCGTCATTACG
CTTGATGTGGTTCTAAGGAGCTACGAGAGGTATTGCTACGGCAACCCAAAGCAAGCCCCTAGCCTAACACGT
TTCCTGAGGATGTTGACAGATCTGACACTCGTTGGATTGACCGTAAGGCGCCTTTCACGCTTCTTGGACAATG
GAATTATCGCAAACGTCGAGGACTTCGTACCAATCGGACACAGAAAGCCAACCATCTACGAGAAGTTCATG
ATGGTTGTACAACGTCGCTACGCTGGGTTGATAGCGTACGTTCTACGTATCCTGGGGAAGTACAACATCTGCA
ATGTTAGTATGTTGATCGAGAAGGCAACTACACGCAACCCTGATTTACGTAAGGACTTATTGCGTACGACCTC
ACAGATACGTGCTTTCCCGATGGTCAGCGCTGGTATTCACAGCCACCCAAACTCTGCCATGTGGCGATCAGCA
ATGGCGGCACACATGAACAAGATAGCCGAAGAAGCAGGCTACATACCATACAACGTCTCGAAATCAGCTAA
TGATAATGTTGGTACAAGATACTTCTACTTCATGAAAGATCTTGCACAGAAGTACGAAGACCCTGAATTACCA
CACAATGCAGCTATCATCATGACTGATGTTGATTACTATGCCGATATGAATGCATGGTTACTCACAGAGAAAC
CAATCTTGATCTATACTCTCTATCCTACTACAGCAGCTCATAGGAACGATGAGTACTCATTCTACATCGATCC
AGCTACGAATTCAGTGGTCTACAACGTATCAGGAGGTGCTAAGTATCAGCACCACCTATGGGACTACAAGGG
TGATACTATCGGAACATTCGACCGCAAGTGCGACTTGATCACTTACTCAATCGAACAACGGAAGATCGCAGG
TGACGAGAACCACAGGTTCATCCTCCTATGTCCAGTGGCCAAGGTACACCACCCATACGGCTTCCTCGTCGGT
ACTGGAGCTTGGGGACTACAACGGCGAGACTTGACCAGTGGTTACTTGTACGATGACATCACCGACCAGTTG
TCGATCTTAGTTAGTGAGCAACATTCAGTAGAGATCACTGGCACTTTGTACGAATCAATCAAGCAACGATTGC
TGAACAAGACAGCATCACCATTGGTCTCTGACATTGAACGCCTGTTGCGAGACAACAAGGTGACAGAACAC
GCTCTGATTGCACCCTTGCTATACAATCACATGGGACTAGAGTTACATCGTAACGTGGTCCACACAACAACAT
TGACCACTAACTACCATCCCGTTGAACTTGGCGCTCTTGCTACCGAAGATGGTAAACCAGGAGGTGAAGCAG
GACACACGACGCTTGTCAATCCAGGCGCTTGCTTCCCAACAGTAAGCGAAGCAAGTGACATCTCAACAGTCA
TACACCGCGTTCTCAAGCCTTGCAACACCATTACTCCACCTCGCATCTACAACAACTATGCCGATGAATTCAG
GAAGATCTTACTGATGGGAAAGGAACGTATGGGTGCCCCATGGAGTTACCCCCAAGTCATTGCTGAACAGAA
TGGCCCTCTACAACGAGTACGAATCGACCAGATCCGACACACCGTAATGCTCAATTCTCCGAACCGTAACAG

TGCCTTCGTCAAGAACGAAGCGTATCCAACTATCAACGCACCACGGAACATCACCACAATGTCGGGAGAGG
TAACGTTGAGTCTCTCCAGCTTCACGTACCCTTTCAAGCACGAGATCTTGAAGAACCACAAGACTAAGTTCTA
CTCACCAGGCATGTCACCAATCGAGATCACAGAACGACTAGCCGAACTGACAACCACAGATGGTGTAATAG
CCACTGATTACACAAAGTTTGATGGCTCCATATCACAATGGCTGCAACTGTTCGTCAAGAACAACTATACAGC
TTGGCTAGATCAGAGATACGTAGGTGAGTTCGAACACTGGTTCAATCAGGTGTTCGTCCAAAGGGCTCGTACT
AAACATGGAGTGCAGTACAATCCTCATTGGTCAACACGCAGTGGAAGCCCACTCACAACTGATGGGAATACT
CAGATAAATGCTTTTGTTGTCTACTGCGCCCTCCGCAAACTGAAATACGAGCCCAAAGAGTCGTTCAGCAAG
CTCGGCCTCTACTGTGGTGATGATGGTTATACACGCTACTATCCCGGTCTCGACCTCATGATGACAACAGTTG
CGAAGGAACTCGGCCTTACCCTTGATATTGAGGTGAACTTCCACGGGCCGTACAGCTTTTGTGGTAGACGGTT
CATAGATCCATCACGTATTCCTGACAGCTATCAAGACATAAAGAGAACTCTGCCGAAGTTGCATCTGGTACG
TAATGGGACCATGTCAATTGCACAGAGATTGACGAACAAGGCTGTTGGATACCAGACAACCGACGACAAGA
CGCCCATCATCGGTAACTGGGCACGTAAGGTGCTTGCGTTAACAGGATTGCAACCCAGAAACTTGTCTCATG
AAGAAGCTTACAAGATGAATCAACCATGGCCTCAAGAATCTGAACTTGAAATCGAAGAACACGTAGCAGCT
GACTTGGAGATCACTAGTCAAGAACTTAAGCAACTATGCGACTACATTGATCAGACTACATCACTAGATGAC
TTTCCAATCCTGTTCGAATGGACACCTGATGCTAAGATAACAGCCGTCAGAAGTGGGGTAATTGAGGCCGTTC
ATATTAATGAACAAAACAATCAACATGGCGGAAAACGTGATCAAAGCACTTCAAGACCAAGTCGACAAGCT
CAAGATGGCAGAAGACAAAGGCGATCTACGTCAGCTCACCAACAATCAAGCCGAACTACTGGTCGCCCACT
CGCAATGGCTACAGGGGGTTCCAGGAGCCCTAGCATATCAAGTCTCAGCTCTAACCAAACGAGCCCAGAAAT
TCAACGCAAAGCGCGTGGCGCTAGGGCTAGAAGCAAACGAATTGGACACTTCAGTGATCCTGAACTTGCAAT
ACGCCCTGACAGCAGCGATAGTCGGAGCCAACCGGGACGGGTTAATAATGATGTTAGCACCACTCTTTCGAG
AGGAACCCTCGTGCTCTCACGAACCCCAAAGCAGCAAATCCAAACGAAGGTTTGGGAAGCTACCGCACGAG
AATGA

Figure S2. Sequences of optimized RNLV1 ORF1a.

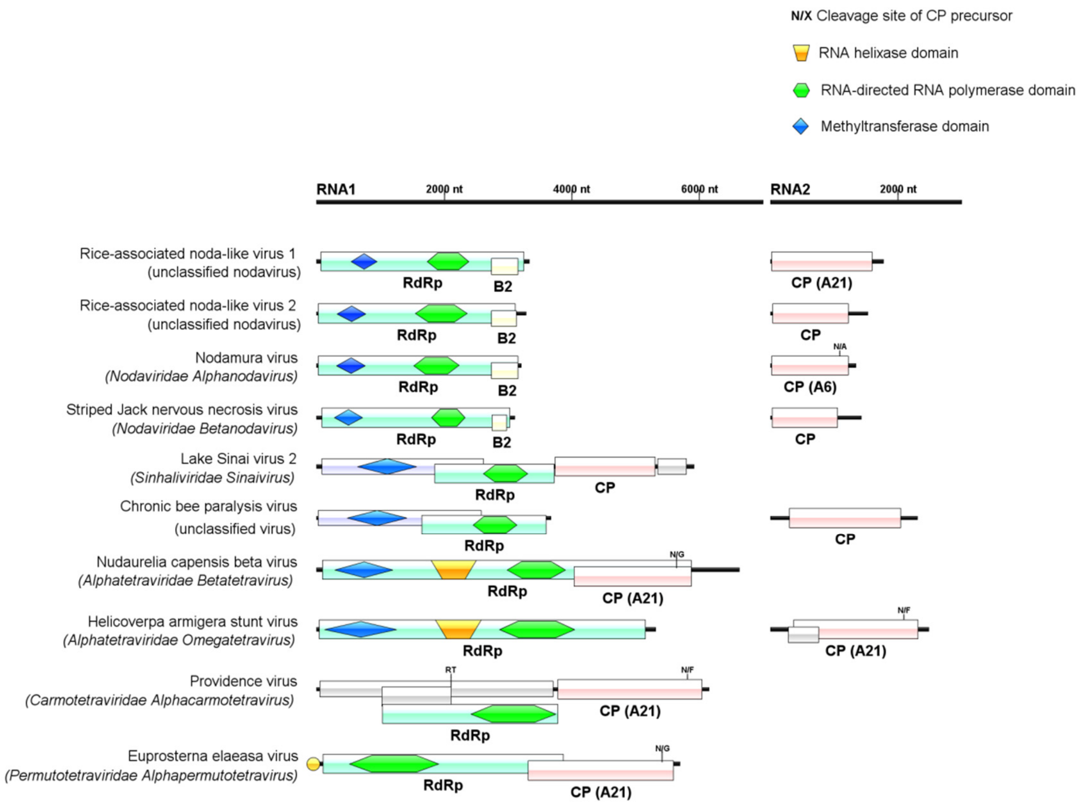

Figure S3. Genome and encoded protein comparisons of RNLV1, RNLV2 and related virus representatives. The name and current taxonomy of the viruses were listed on the left. Viral genome is represented by black lines. Viral proteins are represented by colored rectangles and predicted domains (by CD Search) are represented by other shapes. Viral proteins from different genera and domains that function identically are marked in the same color.
